# Pharmacometric Modeling of the Impact of Azelastine Nasal Spray on SARS-CoV-2 Viral Load and Related Symptoms in COVID-19 Patients

**DOI:** 10.3390/pharmaceutics14102059

**Published:** 2022-09-27

**Authors:** Christiane Dings, Peter Meiser, Frank Holzer, Michael Flegel, Dominik Selzer, Eszter Nagy, Ralph Mösges, Jens Peter Klussmann, Thorsten Lehr

**Affiliations:** 1 Department of Clinical Pharmacy, Saarland University, 66123 Saarbrücken, Germany; 2 Saarmetrics GmbH, Starterzentrum 1, Universität des Saarlandes, 66123 Saarbrücken, Germany; 3 URSAPHARM Arzneimittel GmbH, Industriestraße 35, 66129 Saarbrücken, Germany; 4 CEBINA GmbH, Karl-Farkas-Gasse 22, 1030 Vienna, Austria; 5 Faculty of Medicine, Institute of Medical Statistics and Computational Biology (IMSB), University of Cologne, Kerpener Str. 62, 50937 Cologne, Germany; 6 Faculty of Medicine, Center for Molecular Medicine Cologne (CMMC), University of Cologne, Robert-Koch-Str. 21, 50931 Cologne, Germany; 7 Department of Otorhinolaryngology, Faculty of Medicine and University Hospital Cologne, University of Cologne, Kerpener Str. 62, 50937 Cologne, Germany

**Keywords:** COVID-19, SARS-CoV-2, azelastine, PK model, virus kinetic model

## Abstract

The histamine-1 receptor antagonist azelastine was recently found to impact SARS-CoV-2 viral kinetics in a Phase 2 clinical trial (CARVIN). Thus, we investigated the relationship between intranasal azelastine administrations and viral load, as well as symptom severity in COVID-19 patients and analyzed the impact of covariates using non-linear mixed-effects modeling. For this, we developed a pharmacokinetic (PK) model for the oral and intranasal administration of azelastine. A one-compartment model with parallel absorption after intranasal administration described the PK best, covering both the intranasal and the gastro-intestinal absorption pathways. For virus kinetic and symptoms modeling, viral load and symptom records were gathered from the CARVIN study that included data of 82 COVID-19 patients receiving placebo or intranasal azelastine. The effect of azelastine on viral load was described by a dose–effect model targeting the virus elimination rate. An extension of the model revealed a relationship between COVID-19 symptoms severity and the number of infected cells. The analysis revealed that the intranasal administration of azelastine led to a faster decline in viral load and symptoms severity compared to placebo. Moreover, older patients showed a slower decline in viral load compared to younger patients and male patients experienced higher peak viral loads than females.

## 1. Introduction

Since the emergence of SARS-CoV-2 in December 2019, treatment options for coronavirus disease (COVID-19) have been scarce. Hence, many previously approved compounds have been reassessed for drug repositioning regarding the treatment of acute COVID-19. Several potential candidates could be found in a retrospective study that used data mining of electronic health records and could associate the usage of several common antihistamines with lower incidence of SARS-CoV-2 infections [1]. One of the identified compounds was the histamine-1 receptor antagonist azelastine hydrochloride. Azelastine has been approved for the treatment of seasonal or perennial allergic rhinitis for more than 30 years. An azelastine nasal spray formulation is currently available at a concentration of 0.1% *w*/*v* that is known to be well-tolerated, with bitter taste and somnolence being the most common side effects [2]. Patients older than 60 years with a history of previous azelastine use showed a significantly lower rate of SARS-CoV-2 infections compared to patients without previous azelastine treatment (odds ratio 0.41, 95% CI 0.25–0.68) [1].

Independently, in vitro studies have shown that azelastine inhibits the interaction of the SARS-CoV-2 spike protein with the ACE2-receptor (EC50 = 3.834 µM) in a pseudo-virus infection assay [3]. Tested for its direct antiviral activity on the infection of Vero E6 cells with SARS-CoV-2 isolate USA-UF-1/2020, azelastine was effective with an EC50 of 2.24 µg/mL [1]. Furthermore, daily 20 min treatment with 0.02% azelastine solution in assays with reconstituted human nasal tissue showed antiviral activity by reducing the viral particle numbers by >99.9% 48 h post infection [4].

Recently, the CARVIN study was performed to evaluate the effect of azelastine nasal spray on the SARS-CoV-2 viral load and infection-related symptoms in SARS-CoV-2 positive patients with mild symptoms who do not require inpatient treatment [5]. In this double-blind study, patients received either placebo, 0.02 or 0.1% azelastine nasal spray three times daily for 11 days of treatment. COVID-19 related symptoms were reported daily, and SARS-CoV-2 viral load was assessed from nasal swabs on seven occasions throughout the study. Here, after treatment, virus load was reduced in all groups (*p* < 0.0001) but was greater in the 0.1% group compared to placebo (*p* = 0.007). In a subset of patients (initial Ct < 25) viral load was strongly reduced on day 4 in the 0.1% group compared to placebo (*p* = 0.005). Negative PCR results appeared earlier and more frequently in the azelastine treated groups: being 18.5 and 21.4% in the 0.1 and 0.02% groups, respectively, compared to 0% for placebo on day 8 [5].

However, analyses of the underlying mechanisms and dose-effect relationships considering individual infection characteristics were missing. Here, mathematical mixed-effects modelling can be beneficial as variability between patients, such as different baseline viral loads, can be considered. Furthermore, the relationship between the local drug concentration, drug effect, viral replication and the occurrence of virus-related symptoms is complex and driven by non-linear time-dependent processes. Hence, mathematical modelling using non-linear mixed-effects techniques can provide meaningful insights regarding the underlying mechanisms.

This work has aimed to use non-linear mixed-effects modelling for (i) developing a pharmacokinetic (PK) model for azelastine, (ii) describing the effect of azelastine on viral load and symptoms of SARS-CoV-2 positive patients from the CARVIN study and (iii) exploring the impact of the covariates on viral load, infectivity, and symptoms.

## 2. Materials and Methods

### 2.1. Studies Included in the Analysis

A PK model for azelastine was developed based on digitized literature data from 5 studies including data after nasal and oral application of azelastine [6,7,8,9,10] since no azelastine plasma concentrations were measured in the CARVIN study. The study was registered in the German Clinical Trial Register (DRKS-ID: DRKS00024520; Date of Registration in DRKS: 12/02/2021) and the EU Clinical Trials Register (EudraCT number: 2020-005544-34). For data digitalization, GetData Graph Digitizer (http://getdata-graph-digitizer.com, accessed on 28 August 2020, version 2.26.0.20) was used according to best practices proposed by Wojtyniak and coworkers [11].

To analyze the impact of azelastine on viral load and COVID-19 symptoms, clinical data from the double-blind CARVIN study were used [5]. Here, 90 COVID-19 patients were included that were recently tested positive for SARS-CoV-2 not requiring hospitalization nor being at risk for severe disease. Patients were randomized into one of three treatment groups: 1 mg/mL nasal spray (0.1% azelastine), 0.2 mg/mL nasal spray (0.02% azelastine) or placebo nasal spray. Treatment was applied as one puff per nostril three times a day.

For the assessment of viral load, daily quantitative PCR (qPCR) was performed from nasopharyngeal sampling swabs on days 1 to 5 and on days 8 and 11. For our analysis, patients were excluded when SARS-CoV-2 qPCR test results were negative for ≥5 of 7 measurements (*n* = 8). Single viral load measurements were excluded if the gene copy numbers of the two measured genes (E and ORF1a/b gene) differed by more than two orders of magnitude. Our modeling analysis was performed using the measurements of the ORF1a/b gene.

During the study, patients had to document the severity of twenty COVID-19 related symptoms (anosmia, ageusia, cough, sore throat, shortness of breath, coryza, general weakness, headache, aching limb, loss of appetite, pneumonia, nausea, abdominal pain, vomiting, diarrhea, conjunctivitis, rash, lymph node swelling, apathy, somnolence) daily from day 1 to 11 on a scale from 1 (very weak) to 5 (very strong). For an individual score of the disease severity, the sum of symptom scores was used as the outcome measure (symptom sum score). Further details of the study are published elsewhere [5].

### 2.2. Data Analysis

Model development and simulations were performed using non-linear mixed-effects modeling implemented in the software NONMEM (version 7.4.3, ICON Development Solutions, Ellicott City, MD, USA). The objective function value (OFV; −2 log likelihood), the precision of parameter estimates assessing the respective relative standard errors (RSE) [12], as well as visual inspection of the goodness-of-fit plots and conditional weighted residuals (CWRES) vs. time [12], were used as evaluation criteria for model selection. A new model was accepted if the addition of one parameter led to a reduction of the OFV by at least 3.841 (chi-square distribution with 1 degree of freedom, α-level 0.05). Dataset generation and graphical visualization of NONMEM results were performed using R (version 3.6.3, The R Foundation for Statistical Computing, Vienna, Austria).

### 2.3. Model Development

First, a PK model describing the plasma concentration-time profiles for azelastine after intranasal and oral application was developed (PK model A) based on literature data from 5 clinical studies [6,7,8,9,10]. Here, one-, two-, and three-compartment models were tested. To describe the absorption process, zero- and first-order processes as well as delayed and parallel absorption were evaluated. For the elimination processes, first-order as well as saturable processes were tested. Since it was anticipated that the local, intranasal azelastine amount is of importance for the description of the effect on the viral load determined by nasal swabs, a second PK model (PK model B) was established to simulate the local intranasal azelastine amount based on prior knowledge about the kinetics of intranasally applied drugs from the literature.

For the description of the change of individual SARS-CoV-2 viral load over time, several known virus kinetic models were evaluated [13,14,15,16]. The influence of estimated azelastine plasma concentrations (PK model A) and estimated intranasal azelastine (PK model B) on different parameters of the virus replication model was evaluated using linear, Emax and hill effect models.

For the description of the symptom sum score, linear and turnover models were tested and linked to the PK-virus kinetic model. Since the symptom sum score comprises 20 symptoms and individual scores ranged between 1 (very weak) and 5 (very strong), a symptom sum score of 20 was defined as the absence of any symptoms. Hence, the model predicted symptom sum score was calculated with an offset of the minimal score of 20.

Covariate analyses were performed for the virus kinetic model and the symptom score model. For this, age, weight, height, BMI, sex, baseline copy number, baseline outcome level and date of initial positive SARS-CoV-2 test were evaluated as covariates. The covariate analysis was performed using forward inclusion (*p* < 0.05) and backward elimination (*p* < 0.001) with each covariate being evaluated univariately (one-by-one) [17].

### 2.4. Simulations

Using the final covariate model, simulations were performed to illustrate the effects of different azelastine treatment schedules and the influence of the covariates age and sex. Here, an average patient was assumed (male, 32 years, treatment with 3 × 0.1% azelastine) and one covariate was varied at a time. For age, simulations were performed for the median, 5th and 95th percentile of the study population (32, 19 and 57 years). Next to the study treatments (placebo, 3 × 0.02% azelastine, and 3 × 0.1% azelastine) 5 × 0.1% azelastine was simulated to explore the benefits of a more frequent application of azelastine.

Furthermore, the impact of preventive treatment with azelastine was explored by simulating the viral load with azelastine therapy starting before the time of infection using 3× and 5× daily application of 0.1% azelastine. With the equation proposed by Goyal et al. [18], the transmission risk T was approximated as a function of the viral load V as

T = (V^α^/(V^α^ + λ^α^))^2^, (1)

with λ representing the viral load at which the transmission risk is 25% (λ = 10^7^ cp/mL) and α representing the slope (α = 0.8), as described by Goyal et al. [18]. To evaluate the impact of the changed transmission risk by azelastine treatment, the difference of the area under the curve (AUC) of T until the time of diagnosis (and presumably isolation of the infected person) was calculated. This difference in the AUC of the transmission risk was used to estimate the spread of SARS-CoV-2 infections based on one index case. For this, the R(t) of a susceptible-infected-removed (SIR) infectious model was decreased by the respective, estimated AUC decrease in the transmission risk. For treatment with placebo, an R(t) of 1.2 was assumed, which represents the reported median R(t) value in Germany for the first two years of the pandemic (2 March 2020–2 March 2022) whenever R(t) was >1 according to the RKI nowcasting [19].

## 3. Results

### 3.1. Pharmacokinetic Model

The digitized PK dataset included 8 mean plasma concentration-time profiles after intranasal application of 0.28, 0.548, 0.55 and 0.822 mg azelastine [6,7,8,9] and 2 profiles after oral application of 2 mg azelastine [10] from five different studies. A summary about characteristics of the study populations is presented in Appendix A Table A1. In total, the dataset contained 130 azelastine observations after 10 dosing events.

**Figure A1:**
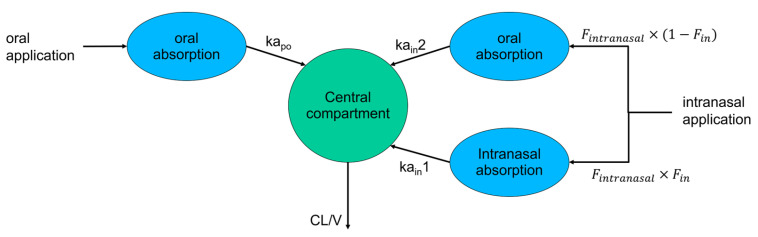
Schematic representation of the azelastine PK model A describing the plasma concentration-time profiles after oral and intranasal applications.

**Figure A2:**
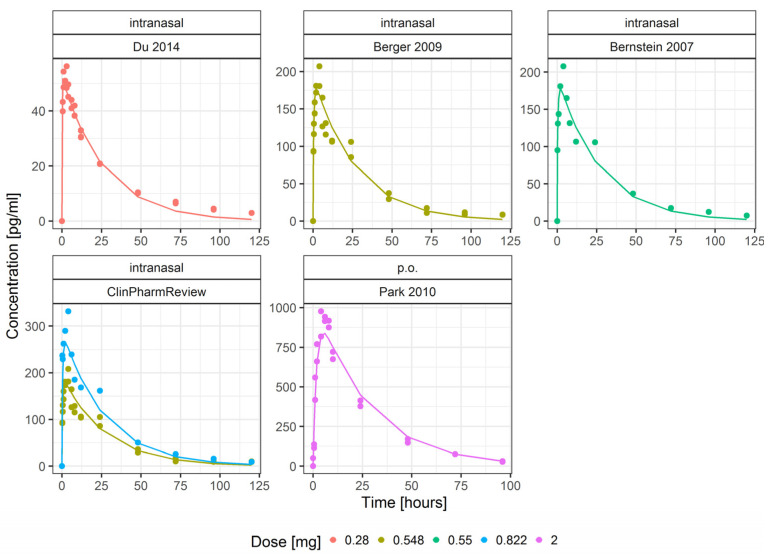
Azelastine plasma concentration-time profiles after intranasal or oral application of various amounts. Points represent observations from 5 different studies [6,7,8,9,10] and lines represent model predictions of the PK model A. Colors indicate dose amounts.

**Figure A3:**
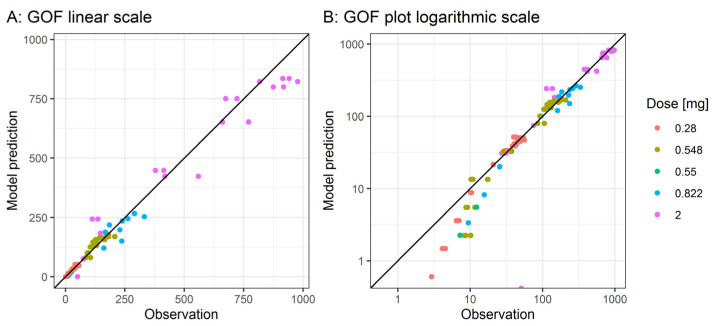
Goodness-of-fit (GOF) plots for PK model A showing predicted vs. observed azelastine plasma concentrations on linear scale (**A**) and log-log scale (**B**). Black lines indicate the lines of identity.

The azelastine plasma concentrations were described best by a one-compartment model with parallel absorption via a fast and a slow absorption arm after nasal application of high volumes of spray (i.e., two puffs per nostril, 140 µL/puff, PK model A, Appendix A Figure A1). For small volumes (one puff per nostril, 140 µL/puff), the fraction absorbed via the fast absorption arm was estimated to be approximately 100% and, thus, was finally fixed to 100%. The absorption rate for the fast arm was estimated very high and fixed to 100 h^−1^. The relative bioavailability of intranasal absorption was estimated to be 36.8% compared to the oral bioavailability. Parameter estimates of the final PK model A are listed in Appendix A Table A2. Model predictions versus time and goodness-of-fit plots are presented in Appendix A Figure A2 and Figure A3. Except for very low concentrations, all data points were randomly spread around the line of identity indicating good descriptive performance.

**Figure 1:**
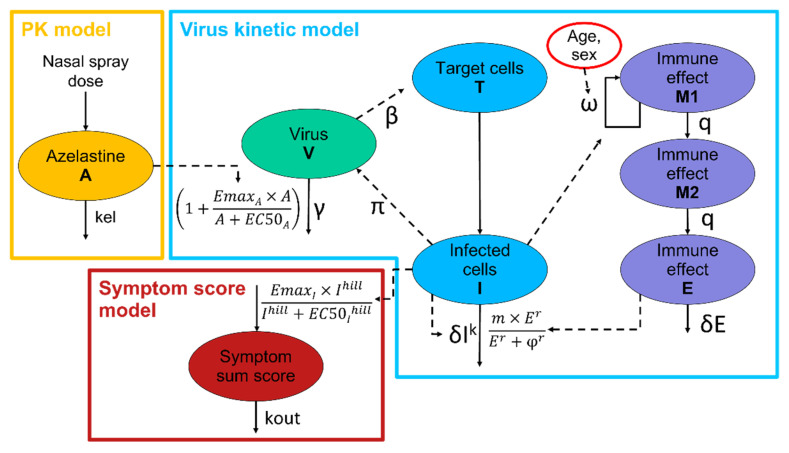
Schematic representation of the final PK-virus kinetic-symptom score model. The PK model is represented in yellow. Different parts of the virus kinetic model are represented in green, blue and violet. The symptom score model is represented in red.

The second PK model (B) was established based on literature knowledge about the kinetics of intranasally applied drugs to simulate the local intranasal azelastine amount. In this model, the amount of azelastine in the respective spraying formulations was administered as a bolus amount and cleared with an elimination half-life of 20 min (t_1/2_ = 0.0138 days corresponding to an elimination rate k_el_ of 49.9 day^−1^, Figure 1) as described by Schipper et al. [20].

### 3.2. PK-Virus Kinetic Model

From the 90 patients included in the CARVIN study, 8 patients were excluded due to negative SARS-CoV-2 PCRs on ≥5 of 7 measurements, resulting in the final virus kinetic dataset of the CARVIN study containing 565 viral load qPCR measurements from 82 patients. Subjects included in this analysis were on average 32 years old (range 19–60 years) and weighed 72 kg (range 50–129 kg). Forty-two patients (51%) were female. One patient reported fever at baseline, one patient has been previously infected with SARS-CoV-2 and three patients had previously been vaccinated. The median delay between positive PCR test result and study inclusion was 1.44 days. Table 1 shows that the treatment groups had comparable demographic characteristics.

Regarding the azelastine nasal-spray application in the CARVIN study, no exact dosing times were recorded. The study protocol suggested two puffs (one puff per nostril) three times a day (after waking up in the morning, around lunchtime and in the evening), preferentially every 6–8 h. Adherence concerning morning, midday and evening doses was self-reported by the patients together with the symptom questionnaire. For our analysis, the applications were assumed to be administered at 08:00 (morning), 14:00 (midday) and 20:00 (evening) if the patient self-reported the application of the respective dose.

The semi-mechanistic model by Goyal et al. [14] (Figure 1) described the viral load data best in comparison to the tested approaches. Here, target cells (T) can become infected by free virus turning them into infected cells (I). Infected cells produce virus (V) and be eliminated by an immune response. The model includes a fast immune response with a direct effect on the elimination of infected cells and a late T-cell immune response, depicted via transit compartments that describe the delay between infection and T-cell immune response (Figure 1, shown in purple).

Most virus kinetic model parameters were fixed to values reported by Goyal et al. [14] (see Table 2). Viral infectivity (β = 8.89 × 10^−9^ virion^−1^day^−1^), virus elimination rate (γ = 1.92 day^−1^) and the time between infection and diagnosis (ALAG = 6.51 days) were estimated for our study. A moderate IIV of 54 %CV was identified for the time between infection and diagnosis ALAG. All model parameters are shown in Table 2.

The two PK models (A and B) were tested for the impact of azelastine exposure on the virus kinetics. Here, PK model A depicted the plasma concentration after oral and intranasal dosing of azelastine. PK model B was developed to describe the intranasal azelastine amount. Both models were linked with the virus kinetic model and described the azelastine drug effect equally well using Emax effect models with a statistically significant (*p* < 0.05) influence on the virus elimination. However, PK model B was favored due to the principle of parsimony and the local mode of action for azelastine as well as the higher precision of parameter estimates. At maximum effect, azelastine increased the virus clearance by 32.2%.

Age and sex could be identified as covariates influencing the virus kinetics. Older patients showed a reduced late T-cell response to SARS-CoV-2 infected cells with an estimated exponent of −0.287. Female patients had a 95% increased late T-cell response compared to male patients (see Table 2).

**Figure 2:**
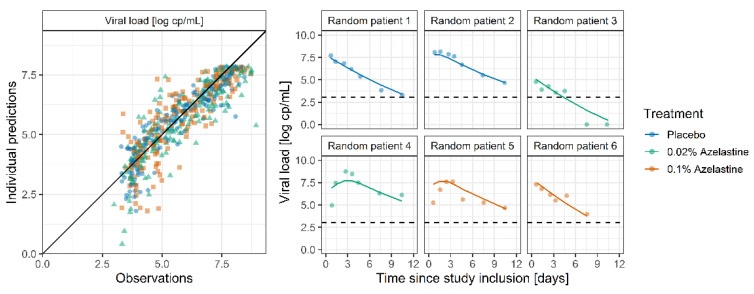
Goodness-of-fit plots for the PK-virus kinetic model (**left**) showing individual predictions vs. observations excluding negative test results (84.1% of which were predicted to be below the lower limit of quantification of 3.02). The black line indicates the line of identity. Exemplary plots (**right**) for two randomly drawn patients per study arm. Points indicate observations. Lines indicate individual model predictions. Observations below the LLOQ were set to 0 log cp/mL. Dashed lines indicate the LLOQ.

The goodness-of-fit plot for the final model (Figure 2, left) shows that the viral load was well described throughout all treatment groups with observations vs. model predictions randomly scattered around the line of identity. Furthermore, Figure 2 (right) shows the good agreement between observed and model-predicted individual viral load-time profiles for two exemplary patients from each treatment group.

### 3.3. Symptom Score Model

The dataset for the symptom score model included 859 observations of the symptom sum score for the 82 patients with viral load data described in Section 3.2. The median symptom sum score at study inclusion was 35. After 11 days, symptoms were considerably reduced with a median score of 23.

For the symptom score model, the parameters estimated for the PK-virus kinetic model were fixed, including the individually estimated values for the lag time between infection and diagnosis. The symptom sum score was described best by a turnover model (Figure 1, red part). An increase in the symptom score was triggered by the number of infected cells implemented by a hill effect model with an EC50 of 5.01 × 10^5^ infected cells and a hill coefficient of 0.298. The estimated elimination half-life of the infected cells was 1.87 days. A high IIV (78.7 %CV) was identified on the maximal impact of the infected cells on the outcomes (Emax). No significant impact of covariates on symptom sum score could be found.

**Figure 3:**
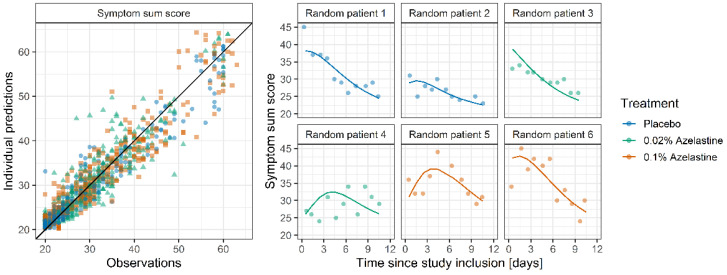
Goodness-of-fit plots for the symptom score model (**left**) showing individual predictions vs. observations. The black line indicates the line of identity. Exemplary plots (**right**) for two randomly drawn patients per study arm. Points indicate observations. Lines indicate individual model predictions.

The goodness-of-fit plot of the final symptom score model (Figure 3, left) shows that the symptom sum score was well described for all treatment groups with all points randomly scattered around the line of identity, even for extreme values with scores of >50. Furthermore, Figure 3 (right) shows the good agreement between observed and model predicted individual symptom sum scores for two exemplary patients from each treatment group.

### 3.4. Simulations

To illustrate the effect of the covariates and different azelastine doses and treatment schedules, simulations were performed with the final PK-viral load-symptom score model. Scenarios for an average patient were tested (male, 32 years, treatment with 3 × 0.1% azelastine) and respective covariates were varied.

**Figure 4:**
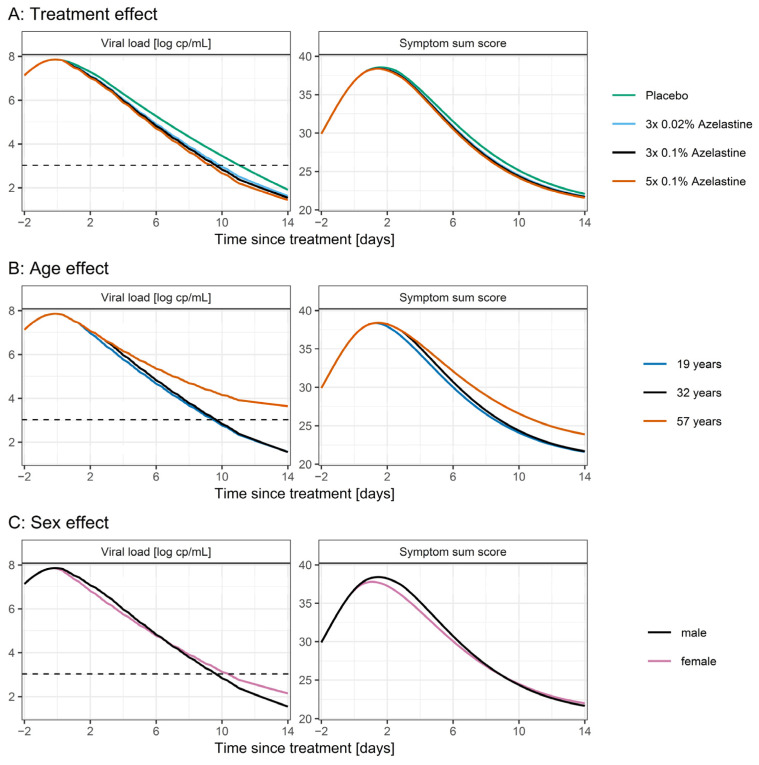
Scenario simulations of the viral load and symptom sum score dependent on univariate changes in treatment (**A**) or the covariates age (**B**) and sex (**C**). Black lines represent the standard patient (32 years, male) receiving 3 × 0.1% azelastine treatment. Colored lines indicate the univariate change of the respective covariates. Dashed lines indicate the LLOQ of the viral load.

Results of the simulations are shown in Figure 4. Patients treated with azelastine showed a faster decrease in the SARS-CoV-2 viral load and symptom sum score compared to patients receiving placebo (Figure 4A): The time until the viral load drops below the lower limit of quantification (LLOQ) was reduced in patients receiving azelastine. Here, the effect size was dependent on dose and frequency of azelastine administration (10.6 days, 9.0 days, 8.7 days and 8.4 days after study inclusion with placebo, 3 × 0.02%, 3 × 0.1% and 5 × 0.1% azelastine, respectively). The time until the symptom score drops below 25 (corresponding to 15 very weak and 5 weak symptoms) was longest for patients treated with placebo (10.1 days) and drops with azelastine treatment to 9.6, 9.4 and 9.3 days for 3 × 0.02%, 3 × 0.1% and 5 × 0.1%, respectively. Figure 4B illustrates the slower decline in viral load and symptom score in older patients: Patients aged 57 years did not reach a viral load below the LLOQ after 11 days. Patients aged 19 and 32 years reach the LLOQ after 9.5 and 9.6 days, respectively. The time until symptom score of 25 was much shorter for younger patients (9 vs. 12 days for patients aged 19 and 32 vs. patients aged 65, Figure 4B). Male patients experienced higher peak symptoms (maximum symptom sum score 38.4 vs. 37.8 for male and female patients, Figure 4C). However, in the terminal phase of the disease after 7 days, male patients showed a faster decline in viral load and symptoms.

**Figure 5:**
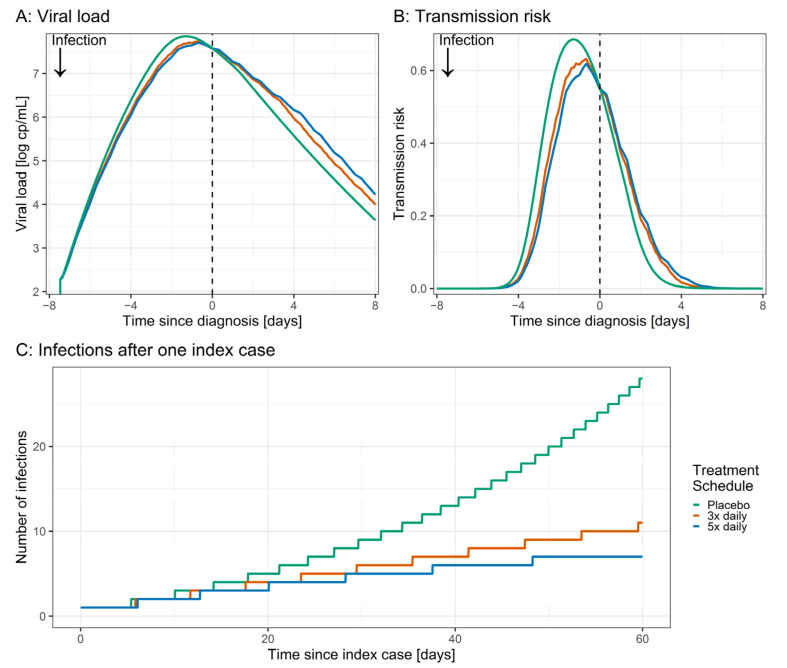
Scenario simulations of SARS-CoV-2 viral load (**A**), transmission risk (**B**) and subsequent infections (**C**) after placebo (green) vs. preventive administration of 3 × 0.1% azelastine (red), 5 × 0.1% azelastine (blue). Simulated preventive treatment started 11 days before infection and was continued until the end of simulations. (**A**) Viral load after infection with SARS-CoV-2. (**B**) Transmission risk associated with the respective viral load. (**C**) Number of infections resulting from the detection of one index case assuming modest contact restriction measures (baseline placebo R(t) = 1.2).

To explore the possible impact of preventive treatment with azelastine, scenario simulations were performed assuming treatment with three- and five-times daily applications versus placebo before the time of infection. Here, slower increase and lower peak viral load upon azelastine treatment were predicted (Figure 5A). Next, the transmission risk was calculated based on the viral load. For patients receiving three times and five times daily azelastine, the AUC of transmission risk until the time of diagnosis was reduced by 11% and 15%, respectively, in comparison to patients receiving placebo (Figure 5B). For a subsequent scenario, this decrease in transmission risk was used to estimate the spread of SARS-CoV-2 infections based on one index case. Here, with a baseline R(t) of 1.2 for patients treated with placebo, one index case leads to 28 infections after 60 days (Figure 5C). For treatment with azelastine, 11 (R(t) = 1.07) and 7 (R(t) = 1.02) cases could be estimated for treatment with three and five times daily azelastine, respectively.

## 4. Discussion

In this work, pharmacometric modeling was applied to (i) describe the PK of azelastine after intranasal and oral dosing, (ii) adopt a virus kinetic model describing the SARS-CoV-2 viral load and (iii) develop a model describing the symptom severity of COVID-19 patients treated with placebo or azelastine nasal spray.

A one-compartment model with parallel absorption for the intranasal application described the PK of azelastine in plasma best (PK model A). Parallel absorption models have been previously suggested for other drugs after intranasal application [21,22], assuming that part of the dose is swallowed and absorbed via the gastrointestinal tract. In our analysis, the effect of parallel absorption was only observed for the application of two sprays per nostril. With one spray per nostril, the fraction absorbed via the fast and presumably intranasal absorption pathway was estimated to be 100%. This effect might be due to the difference in the total volume of applied spray, where parts of the larger volume of two puffs are swallowed. Overall, PK model A described the data very well with the tendency of slightly underpredicting low concentrations in the final elimination phase. However, as the data were obtained by digitalization of published concentration-time curves, data points might be subject to inaccuracies due to known digitalization limitations in linear scale plots [11]. Nonetheless, the estimated azelastine bioavailability of 36.8% of the nasal spray application and an elimination half-life of 18.6 h are in line with previous non-compartmental analyses reporting 40% bioavailability and a half-life of 22 h [2].

A second PK model was developed describing the intranasal amount of azelastine (PK model B) in comparison to the plasma concentrations of PK model A. In PK model B, the elimination rate was set to correspond to the nasal mucociliary clearance of 20 min as reported by Schipper et al. [20]. The bioavailability was assumed to be 100% following the modeling results from PK model A for which no swallowing of the dose was assumed applying of one spray per nostril. For the description of the effect of azelastine on the viral load, measured by nasal swabbing, PK model B was favored as the descriptive performance of both models was comparable, but model B had a simpler structure with a more plausible local mode of action.

For the description of the virus kinetics in the CARVIN study, a previously published virus replication model was utilized. Here, only two population parameters had to be adapted and estimated for an accurate description of the data. For this, the viral infectivity (β) and virus clearance (γ) parameters were estimated about 85% lower compared to Goyal et al. [14]. This renders the within-host infectivity, which is correlated to the fraction β/γ [23], 18% higher in comparison to Goyal et al. [14]. Furthermore, this change leads to a longer disease duration and a higher peak viral load. Both effects might be attributed to the difference in infectivity and severity between SARS-CoV-2 variants (VOCs) since the model developed by Goyal et al. was based on only data with wild-type SARS-CoV-2. In the CARVIN study, information regarding VOC infections was available for 59 patients (66%) with 92% of these carrying the VOC alpha (B.1.1.7). At the time of data collection (3 March–28 April 2021), VOC Alpha increased from 54.6% (4 March 2021) to 93.9% (29 April 2021) in Germany [24]. Hence, it is reasonable to assume that most study patients (including those with missing VOC infection status) were infected with VOC Alpha and differences in model parameters are due to substantial differences in virus replication kinetics between wild-type and VOC Alpha. Importantly, azelastine’s anti-viral effect was found to be comparable against the D614G variant (one amino acid exchange in the Spike protein relative to the wild-type virus) and Alpha variant in in vitro infection models [4].

The intranasal amount of azelastine had a significant impact on the virus elimination rate with a maximum acceleration by 37% (*p* = 0.0044), as simulated by PK model B. The estimated EC50 of 0.848 μg azelastine corresponds to 0.28 mL of a 7.24 μM formulation. In previous in vitro studies, azelastine inhibited the virus entry into the cell by blocking the binding site of the SARS-CoV-2 spike protein with an EC50 of 3.834 μM [3] and EC50 of 2.2 to 3.7 µM in pre-infection and 4 to 6.5 µM in post-infection treatment settings in infection assays with different variants [4], which is in line with our findings.

The model was used to simulate the study treatment (placebo, 3 × 0.02% azelastine and 3 × 0.1% azelastine) as well as treatment with 5 × 0.1% azelastine. Analysis of the simulation results revealed a beneficial effect of all applied doses vs. the application of placebo with the largest effect being observed at 5× daily application of 0.1% azelastine.

As the analysis of PK model A revealed a higher systemic bioavailability after oral compared to intranasal applications, a dedicated investigation of potential (systemic) treatment benefits of oral administrations in COVID-19 patients could be reasonable. However, this analysis was only based on data after intranasal application and viral load measurement from nasal swabs. Hence, it was not possible to differentiate between local effects and potential systemic effects. Furthermore, it might be unreasonable to assume that oral administration of azelastine, concentrations in the target sites of SARS-CoV-2 would be large enough to observe a treatment effect.

Age and sex could be identified as covariates on the extent of the late T-cell immune response of the virus kinetic model, which leads to male and older patients experiencing higher maximum viral loads and symptoms. This is in line with previous findings where female patients and younger patients showed a more robust T-cell immune response to the infection with SARS-CoV-2 [25]. Furthermore, older patients showed a slower decline in the viral load, which can be explained by the ageing of the immune system resulting in diminished antibody maturation and more hyperinflammatory and pathological innate responses to SARS-CoV-2 [26,27].

The symptom sum score was described best by a turnover model induced via hill effect to the number of infected cells estimated by the virus kinetic model. The combination of hill effect and turnover model causes the time of the maximum symptom score to be delayed by 1.7 days in comparison to the maximum viral load and by 2.2 days in comparison to the maximum number of infected cells. The IIV of symptoms between patients was very high (58 %CV) and could not be explained by any of the tested covariates. Effects of age, sex and applied azelastine dose on the symptom score were driven by the difference in infected cells estimated by the virus kinetic model. As expected by the mode of action, no additional effect of azelastine on the symptom score could be observed.

Azelastine nasal spray 0.1% has been approved for the treatment of seasonal allergic rhinitis and nonallergic vasomotor rhinitis for more than 30 years. Moreover, since then, its safety and tolerability have been proven in various clinical studies [2]. The most common reported adverse effect of the nasal spray formulation is bitter taste, which can be reduced by improving the dosing technique [8]. Further reported adverse effects were transient and of mild-to-moderate severity including somnolence, nasal burning, and headache [2]. Due to the favorable safety profile and the positive results from retrospective studies on the mined electronic health records [1], an investigation of the use of azelastine nasal spray for the prevention of infection with SARS-CoV-2 is reasonable. Hence, the developed virus kinetic model was applied to simulate the impact of azelastine intranasal application at the time of infection on the spread of the virus. Here, azelastine lowered the peak viral load from 7.08 × 10^7^ (placebo) to 5.37 × 10^7^ and 5.13 × 10^7^ cp/mL for applications of 3 × 0.1% and 5 × 0.1% azelastine, respectively. Assuming the timepoint of diagnosis remained unchanged despite the lower viral load, the transmission risk until diagnosis was mitigated by preventive administration of azelastine, which substantially lowered the number of infected subjects by more than 50% in our scenario (28 vs. 11 vs. 7 after 60 days without, with 3 × 0.1% and 5 × 0.1% preventive azelastine applications, respectively). However, these calculations are based on some strong assumptions. For one, the simulation of the preventive effect of azelastine is based on model extrapolations outside of the data domain. Furthermore, it is unclear whether the time of diagnosis might be altered with lower viral loads. Additionally, the only preventive effect included in the simulation was the lower transmission risk from the infected person to a susceptible individual. However, previous studies also found a lower risk of infection for susceptible individuals taking azelastine [1]. Hence, further clinical studies would be needed to evaluate the preventive potential of azelastine nasal spray. For the treatment of acute disease, a higher frequency of application (5 × 0.1% vs. 3 × 0.1% azelastine) shortened both the time until symptoms resolved (symptom sum score) as well as the time until viral load dropped below the LLOQ. In the preventive treatment scenario, model simulations of both dosage regimens also favored a higher azelastine dosage frequency to minimize transmission risk.

## 5. Conclusions

Mathematical models were developed to describe the PK of azelastine and the effect of azelastine vs. placebo nasal spray on SARS-CoV-2 viral load as well as COVID-19 symptoms. The PK of azelastine after an intranasal application was best described using a parallel absorption model if two sprays per nostril were applied, implying that parts of the larger applied volume of the formulation (2 puffs) are swallowed. Furthermore, modeling revealed that the intranasal azelastine amount has a significant impact on the viral load measured by nasal swabbing in patients with mild disease. In our analysis, the number of infected cells triggered the disease symptoms with the peak of symptoms being delayed by 1.7 days in comparison to the peak viral load. The impact of azelastine on the viral load also translated to a significantly lower symptom score in patients treated with azelastine in comparison to patients receiving placebo nasal spray. Furthermore, the age and sex of the patients had a significant impact on the viral load with older patients showing a slower decrease in viral load in comparison to younger individuals and male patients experiencing higher peak viral loads compared to females.

## Figures and Tables

**Table 1:** Summary of the study population used for model development of the PK-virus kinetic and symptom score models. Age and weight are summarized as medians and standard deviations (sd). Patients’ sex is summarized as the percentage and number of male patients.

Group	*n*	Age [Years] (sd)	Weight [kg] (sd)	Sex [% male] (*n*)
Placebo	27	33 (13.6)	70 (16.3)	48.1 (13)
0.02% azelastine	28	28 (12.8)	70.5 (15.7)	53.6 (15)
0.1% azelastine	27	35 (13.1)	75 (15.7)	44.4 (12)
All	82	32 (13.1)	71.5 (15.8)	48.8 (40)

**Table 2:** Model parameters of the final PK, PK-virus kinetic- and outcome model. No interindividual and residual variability was estimated for the PK model due to lack of PK data.

Parameter	Value (RSE ^1^/Shrinkage) *	Unit	Source	Parameter Description
**PK model**				
k_el_	49.9	day^−1^	[20]	Azelastine elimination rate
**PK-virus kinetic model**				
ALAG	6.51 (3.2%)	days	estimated	Time between infection and diagnosis
β	8.89 × 10^−9^ (1.2%)	virion^−1^day^−1^	estimated	Viral infectivity
γ	1.92 (6.7%)	day^−1^	estimated	Virus elimination rate
δ	3.1	day^−1^cells^k^	[14]	Elimination rate of infected cells
ω	2.75 × 10^−5^	day^−1^cells^−1^	[14]	Extend of T-cell response
π	398	day^−1^	[14]	Virus production rate
k	0.08	-	[14]	Fast immune response
q	2.4 × 10^−5^	day^−1^	[14]	Differentiation rate of T-cells
δE	1	day^−1^	[14]	Elimination rate of T-cell response
m	3	day^−1^cells^−1^	[14]	Maximum T-cell response
r	10	-	[14]	Hill coefficient of T-cell response
ϕ	100	cells	[14]	Half maximum effective effector cell level
I0	1	cells	[14]	Baseline number of infected cells
M0	1	cells	[14]	Baseline number of T-cells effect cells
T0	10^7^	cells	[14]	Baseline number of target cells
Emax_A_	0.37 (2.9%)	-	estimated	Maximum azelastine effect
EC50_A_	0.0629 (5.1%)	µg	estimated	Half maximum effective azelastine amount
Sex—ω	1.95 (9.8%)	-	estimated	Covariate effect of sex on ω
Age—ω	−0.287 (2.9%)	-	estimated	Covariate effect of age on ω
IIV ALAG	58.0 (16.1%/9%)	%CV	estimated	Interindividual variability on ALAG
AE	1.2 (0.8%)	SD, log cp/mL	estimated	Additive residual error viral load
**Symptom score model**				
Kout	0.37 (5.9%)	day^−1^	estimated	Output rate
Emax_I_	15 (8.3%)	-	estimated	Maximum input rate
EC50_I_	5.01 × 10^5^ (9%)	cells	estimated	Half maximal effective infected cells
hill	0.298 (5.9%)	-	estimated	Hill coefficient
IIV Emax	78.7 (9%/3%)	%CV	estimated	Interindividual variability on Emax
PE	10–9 (2.6%)	%CV	estimated	Proportional residual error symptom sum score

^1^ RSE: Relative standard error, * applicable for model parameter estimates.

## Data Availability

The data presented in this study are available on request from the corresponding author upon reasonable request and with permission of URSAPHARM Arzneimittel GmbH. The data are not publicly available due to use under license for the current study.

## Appendix A

**Table A1:** Summary of the studies used for model development of the PK models. Age and BMI are summarized as medians and range if available. Patients’ sex is summarized as the percentage and number of male patients.

Study	Administration	Doses [mg]	*n*	Age [Years] (Range)	BMI [kg/m^2^]	Sex [% Male] (*n*)
Du 2014 [6]	Intranasal	0.280	22	37.4 (21–55)	24.57	54.5 (12)
Berger 2009 [7]	Intranasal	0.548	36	(18–50)	na	100 (36)
Bernstein 2007 [8]	Intranasal	0.550	na	na	na	100
ClinPharmReview [9]	Intranasal	0.548, 0.822	18	(18–50)	na	100 (18)
Park 2010 [10]	po	2	23	23.0 (19–27)	na	100 (23)

na = not available.

**Table A2:** Parameter estimates of the azelastine PK model A. No inter-individual variability (IIV) was estimated, as the mean curves of azelastine plasma concentrations could be described accurately without additional IIV.

Parameter	Parameter Description	Unit	Estimate	RSE ^1^
**Fixed effects**				
V1/F	Volume of distribution	L	1960	3.6%
CL/F	Clearance rate	L/h	72.9	3.3%
Ka_po_	po absorption rate	1/h	0.55	8.7%
F_intranasal_	Intranasal relative bioavailability	-	0.368	4.3%
Ka_in_1	Intranasal fast absorption rate	1/h	100	FIX
Ka_in_2	Intranasal slow absorption rate	1/h	1.75	20.9%
F_in_ (1 spray)	Intranasal fraction absorbed fast (1 spray)	-	1	FIX
F_in_ (2 sprays)	Intranasal fraction absorbed fast (2 sprays)	-	0.492	6.8%
**Residual error**				
Prop. error	Proportional error	%CV	15.0	16.6
Add. error	Additive error	SD; pg/mL	11.0	59.8

^1^ RSE: Relative standard error.
